# The Isolation and Identification of Bacteria on Feathers of Migratory Bird Species

**DOI:** 10.3390/microorganisms6040124

**Published:** 2018-12-05

**Authors:** Antonella Giorgio, Salvatore De Bonis, Rosario Balestrieri, Giovanni Rossi, Marco Guida

**Affiliations:** 1 Department of Biology, University of Naples Federico II, Complesso Universitario di Monte S. Angelo, Via Cinthia ed. 7, 80126 Napoli (NA), Italy; antonella.giorgio@unina.it (A.G.); marguida@unina.it (M.G.); 2 Consiglio Nazionale delle Ricerche, Istituto di Biologia Agroambientale e Forestale, Via Salaria km 29, 300, 00015 Monterotondo (RM), Italy; ardea.rb@gmail.com; 3 Associazione per la Ricerca, la Divulgazione e l’Educazione Ambientale (ARDEA), Via Ventilabro 6, 80126 Napoli (NA), Italy; 4 Freshwater Science Group; Dipartimento di Scienze Biologiche, Geologiche ed Ambientali (BiGeA), Alma Mater Studiorum—Università di Bologna, Via Selmi 3, 40126 Bologna (BO), Italy; giovanni.rossi7@gmail.com; 5 Hydrosynergy S.C.—Environmental Monitoring and Applied Ecology, Via Roma 11, 40068 San Lazzaro di Savena (BO), Italy

**Keywords:** 16S rDNA, end-point PCR, migratory birds, bacteria, feathers, risk

## Abstract

Worldwide, bacteria are the most ubiquitous microorganisms, and it has been extensively demonstrated that migratory wild birds can increase bacterial global scale dispersion through long-distance migration and dispersal. The microbial community hosted by wild birds can be highly diverse, including pathogenic strains that can contribute to infections and disease spread. This study focused on feather and plumage bacteria within bird microbial communities. Samples were collected during ornithological activities in a bird ringing station. Bacterial identification was carried out via DNA barcoding of the partial 16S rRNA gene. Thirty-seven isolates of bacteria were identified on the chest feathers of 60 migratory birds belonging to three trans-Saharan species: *Muscicapa* *striata*, *Hippolais* *icterina*, and *Sylvia* *borin*. Our results demonstrate the possibility of bacterial transfer, including pathogens, through bird migration between very distant countries. The data from the analysis of plumage bacteria can aid in the explanation of phenomena such as migratory birds’ fitness or the development of secondary sexual traits. Moreover, these results have deep hygienic–sanitary implications, since many bird species have synanthropic behaviors during their migration that increase the probability of disease spread.

## 1. Introduction

Worldwide, bacteria are the most ubiquitous microorganisms. Their complex phylogenetic diversity has produced adaptations that enabled the colonization of almost all habitats under every environmental condition [1,2,3]. Numerous bacteria live strictly associated with humans, animals, and plants, playing important roles in their growth, survival, and development [4]. Furthermore, bacteria are a key component of all ecosystems and substantially contribute to their development [5,6]. There are several ways in which bacteria disperse across different environments (i.e., wind, water, particles), with multiple possibilities for interactions between bacteria and ecosystems. As widely demonstrated, wild birds can increase bacterial dispersion at the global scale through long-distance migration and dispersal [7]. The microbial community hosted by wild birds can be highly diverse, including pathogenic strains that can contribute to infections and the spread of disease [8,9,10,11,12]. For instance, migratory birds have been found to carry enteropathogensms, like *Escherichia coli* or *Salmonella enterica*, as well as causative agents of granulocytic ehrlichiosis, ornithosis, or even Lyme disease [13,14]. Indeed, the extensive migration routes of several species can increase the likelihood of the spreading of highly harmful human pathogens, emerging diseases, and infection outbreaks [15]. Therefore, development of an early warning system to elucidate potential reservoirs of human pathogens is of public interest. Within bird microbial communities, feather and plumage bacteria are of particular interest for two reasons: firstly, they can reduce the fitness of birds [16] or influence secondary sexual traits, for example, by altering plumage color [17,18]; secondly, they can easily infect humans, for example, during handling for scientific purposes (e.g., ringing). The plumage microbial community is influenced by several factors that affect birds from birth; some factors are time-dependent (e.g., phenology, annual cycle, and breeding and wintering areas) [19], while others are fixed, like anatomical parts (e.g., venter, dorsum, or tail) or foraging habits (e.g., ground vs. aerial) [20]. The aim of this study, therefore, was to gain additional information on bacteria that inhabit the plumage of wild migratory birds. Our purpose was to evaluate the detectability and presence of pathogens that are potentially harmful to humans and animals, improve the knowledge of potential hazards associated with bird migration, and identify preventive measures for researchers working on migrant birds. To achieve the objectives set out above, two different sampling methods were applied to evaluate whether the sampling protocol could affect bacterial isolation, rather than determining the complete checklist of bacteria hosted by the sampled birds. In order to complete a double evaluation of possible differences using either microbiological or molecular techniques, we then accepted the introduction of possible alterations of the original bacterial communities due to the different cultural media and incubation cycles. Moreover, this approach avoided the necessity of cloning through the isolation of pure culture with microbiological techniques.

## 2. Materials and Methods

### 2.1. Field Sampling

Sampling was carried out in May 2015 in the Southeastern part of Ventotene (40°47’ N, 13°25’ E), a small island (1.3 km^2^) in the Tyrrhenian Sea. Sampling took advantage of ongoing monitoring activities in the local bird ringing station that is included in the ringing site network of the long-term and large-scale ‘Progetto Piccole Isole’ ISPRA (Istituto Superiore per la Protezione e la Ricerca Ambientale) [21]. Sampling focused on long-distance migrants (i.e., birds moving northwards from sub-Saharan Africa [22]) in an attempt to find bacteria carried from as far away as possible (i.e., outside the Mediterranean basin). Several of these species renew their entire plumage (all feathers) in African winter grounds, [23] and then travel rapidly through Africa (over about 6 weeks) [24]. Among those species, we focused on the ones most commonly ringed in Ventotene in May [25]: Spotted Flycatcher (*Muscicapa striata*), Icterine Warbler (*Hippolais icterina*), and Garden Warbler (*Sylvia borin*).

**Figure 1:**
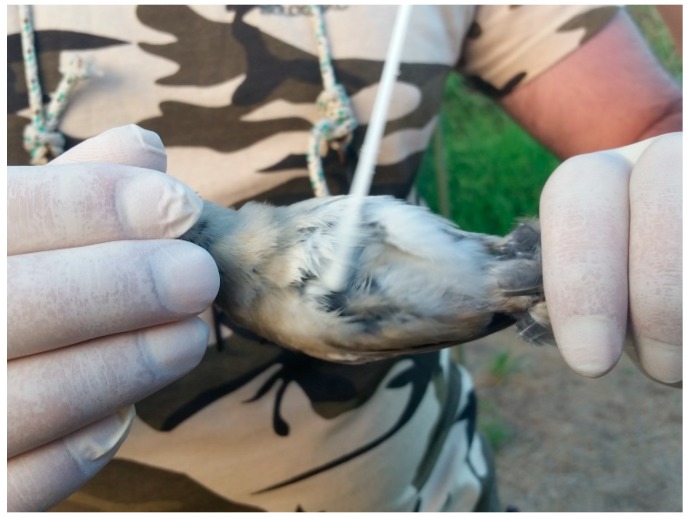
Swab sampling procedure (Ssp).

**Figure 2:**
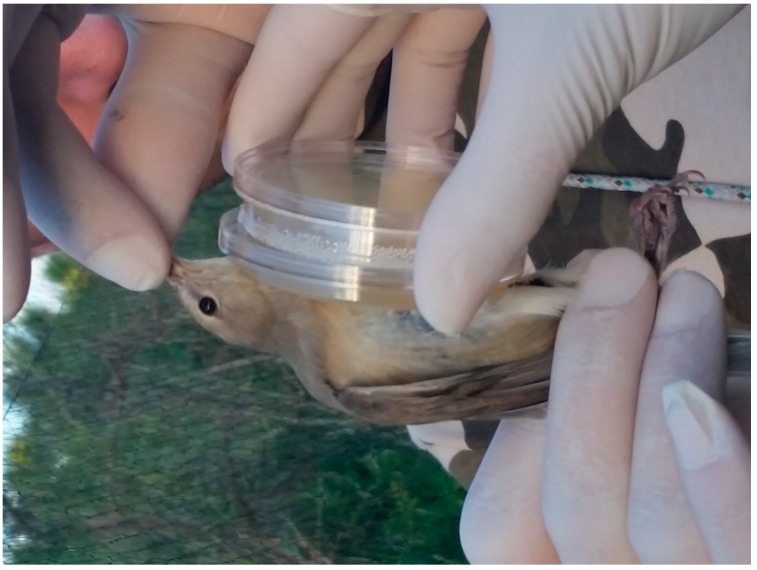
RODAC sampling procedure (Rsp).

Fifty-nine birds were randomly selected after being captured with mist-nets (16 mm mesh size) following the standardized national protocol [21]. Each specimen was removed from the net by one experienced operator who was wearing sterile gloves. The bird was immediately processed with one of two possible microbiological sampling procedures in order to verify whether the sampling protocol followed affects bacterial isolation. The first method (swab sampling procedure—Ssp) involved gently rubbing a moistened swab (Figure 1) wetted with sterile saline solution (0.85% NaCl) several times on the chest feathers in all directions to compressively cover an area of 1.5–2 cm^2^, before placing the swab in a sterile envelope. In the second procedure (RODAC sampling procedure–Rsp), the bird’s chest was placed on a contact plate (RODAC plates) with gentle pressure for 10 seconds (Figure 2). In order to maximize the diversity of bacterial isolates, each sample was collected in duplicate through the use of two media with different cultural characteristic: plate count agar (Oxoid, Thermo Fisher Scientific, Rome, Italy) and Mueller–Hinton agar (Oxoid, Thermo Fisher Scientific). All samples were stored at 4 °C and were transported to the laboratory within 24 h. After microbiological sampling, each bird was transferred to the ringing station, where one expert ringer measured physiological qualitative variables by visually scoring the amount of pectoral muscle and abdominal fat and recording the following standard biometric measures: wing length (±0.1 mm), tarsus length (±0.1 mm), and body mass (±0.1 g) [25]. Every bird was tagged with a unique alpha-numeric ring and was then immediately released.

### 2.2. Laboratory Analysis

Each swab sample (Ssp) was properly diluted, and then one aliquot (100 µL) was plated on each of the two cultural media to isolate the bacteria used in the field for the RODAC sampling procedure: plate count agar (Oxoid, Thermo Fisher Scientific) and Mueller–Hinton agar (Oxoid, Thermo Fisher Scientific). All procedures were conducted in duplicate.

To isolate the mesophilic and psychrophilic bacteria, one sample of each respective Ssp and Rsp inoculated plate was incubated at 37 °C for 24 h, and their duplicates were incubated at 22 °C for 72 h.

After growth, colonies of different shapes, colors, and consistencies were streaked on agar plates to isolate the pure cultures of bacteria for subsequent DNA barcoding identification through 16S rRNA gene amplification. From each subculture, a single colony was picked up and transferred to 100 µL of ultrapure water (BDH Prolabo Chemicals, VWR, Milan, Italy). Bacterial DNA was extracted using the phenol-chloroform method as described by Cheng and Jiang [26]. For the end-point PCR assay, the universal primers complementary to 16S gene regions V3–V6 (about 700 bp) were used: forward V3_F, 5′-CCA GAC TCC TAC GGG AGG CAG-3′, and reverse V6_R, 5′-ACA TTT CAC AAC ACG AGC TGA CGA-3′ [27]. PCR amplification was performed in a Prime Thermal cycler (Techne) using 100 ng of genomic DNA and VWR Taq DNA polymerase (VWR BDH Prolabo Chemicals), according to the manufacturer’s instructions. Initial denaturation at 95 °C for 3 min was followed by 30 cycles of amplification (denaturation at 95 °C for 45 s, annealing at 60 °C for 30 s and extension at 72 °C for 1 min), ending with a final extension at 72 °C for 7 min.

Amplification products were visualized by electrophoresis with a 100 bp DNA ladder (DNA Molecular Weight ladders, Amresco, VWR, Milan, Italy) as a marker under a UV illuminator (VWR UV Transilluminators, Milan, Italy). All PCR products with a desired length of about 700 bp were purified by the QIAquick PCR Purification Kit (Qiagen, Milan, Italy) in accordance with the manufacturer’s instructions. Sequencing was carried out with BigDye Terminator V3.1 (Applied Biosystems, Thermo Fisher Scientific) in accordance with the manufacturer’s protocol and analyzed with the 3130 Genetic Analyzer (Applied Biosystems, Thermo Fisher Scientific). Sequence data were edited using Chromas Lite ver. 2.1.1. software (Chromas Lite version 2.1, Technelysium; http://technelysium.com.au/?page_id=13) and were then compared with those in the GenBank database using the BLAST server (Basic Local Alignment Search Tool; http://blast.ncbi.nlm.nih.gov) hosted by the National Center for Biotechnology Information (NCBI).

### 2.3. Statistical Analysis

A canonical correspondence analysis (CCA) was used to evaluate the occurrence of specific bacterial associations among bird species and to verify correlations among heterogeneous variables. An ANOVA-like permutation test was implemented to assess the significance of the CCA constrained axis. Then, bird biometric and physiological variables were tested to determine if they fit the CCA ordination by employing 9999 permutations and a *p*-value threshold of 0.05. The squared correlation coefficient (*r^2^*) was used to select the best fitting variables. An analysis was carried out through the ‘vegan’ package [28] in the R environment [29].

### 2.4. Ethics Policy

Animal rights. No research was performed on human subjects, and samples were taken by swabbing the animal chest without hurting any animal and no animal research ethics committee prospectively approved this research or granted a formal waiver of ethics approval. A) The study relied on the thirty-year project Small islands led by the Superior Institute for the Protection and Environmental Research (ISPRA) in collaboration with the Migration Museum-Ornithological Observatory of Ventotene and State National Reserve Islands of Ventotene and S. Stefano. These organizations provided the necessary authorizations. B) The research took place in a single sampling site: The three bird species whose plumage was inspected are not listed as endangered by any national or international wildlife conservation program. C) All field sampling procedures are routinely utilized in the ringing station of Ventotene, regulated and approved by ISPRA and did not provide any particular maneuver or stress for birds.

## 3. Results

### 3.1. Microbiological and Molecular Results

We sampled feathers of 59 birds belonging to three trans-Saharan species: *Muscicapa striata* (20), *Hippolais icterina* (20), and *Sylvia borin* (19); for each sample, the used protocol and the results in terms of the number of total colonies and number of isolates of bacteria are shown in Table 1.

The number of colonies observed was comparable among the three species: a total of 362 and an average and median of 18 for *Muscicapa striata*, 383 (total) and 1918 (average and median) for *Hippolais icterina*, and 353 (total), and 19 (average) and 17 (median) for *Sylvia borin.*

The birds with the greatest number of strains were those codified as Ms.1 (30 isolates), Ms.8 (34 isolates), Hi.1 (29 isolates), Hi.18 (31 isolates), Sb.2 (32 isolates), Sb.10 (32 isolates), and Sb.17 (33 isolates). On the other hand, birds with the identification codes Ms.2, Ms.8, Ms.19, Hi.7, and Sb.4 showed the lowest values—8, 7, 8, 8, and 8 strains, respectively.

Concerning the DNA barcoding analysis, a total of 37 different bacteria isolates were identified. Data regarding the number and type of bacteria isolated on each bird are presented in Table 2, Table 3 and Table 4.

Among these, *Bacillus* sp. (65%), *Curtobacterium* sp. (38.3%), *Pantoea* sp. (28%), and *Enterobacter* sp. (25%) were the most frequently isolated genera. *Curtobacterium flaccumfacies* (41%) was the most abundant taxa among isolates, followed by *Pantoea agglomerans* (40%), *Bacillus simplex* (18%), *Bacillus cereus* (16%), and *Bacillus endophyticus* (16%). Table 5 summarizes the results regarding the percentages of the most abundant genera and species in all samples.

The results show some differences in the isolates identified between the samples collected with the two sampling procedures. In *Muscicapa striata*, the taxa *Bacillus endophyticus*, *Bacillus pocheonensis*, *Bacillus simplex*, *Curtobacterium herbarum*, *Klebsiella* sp., and *Pantoea* sp. were only found in samples taken with the swab sampling procedure. No taxa were isolated in samples taken exclusively with the RODAC sampling procedure. On the other hand, *Arthrobacter* sp., *Bacillus flexus*, *Enterobacter hormaechei*, *Enterobacter xiangfangensis*, *Frondihabitans* sp., *Janthinobacterium* sp., *Klebsiella* sp., *Sphingomonas* sp., and *Staphylococcus pasteuri* were found in *Hippolais icterina* only in samples collected with swab sampling procedure, while *Microbacterium pumilum* and *Staphylococcus warneri* were only found in samples measured by the RODAC sampling procedure. Finally, in *Sylvia borin Bacillus pseudomycoides*, *Enterobacter hormaechei*, *Enterobacter xiangfangensis*, *Klebsiella* sp., *Massilia* sp., *Pantoea* sp., *Serratia ureilytica*, and *Stenotrophomonas rhizophila* were found only in samples taken with the swab sampling procedure, while *Bacillus thuringiensis, Brevundimonas nasdae*, *Janthinobacterium* sp., and *Pantoea ananatis* were found only in samples collected with the RODAC sampling procedure. *Klebsiella* sp. was isolated only in samples measured by the swab sampling procedure.

### 3.2. Statistical Results

**Figure 3:**
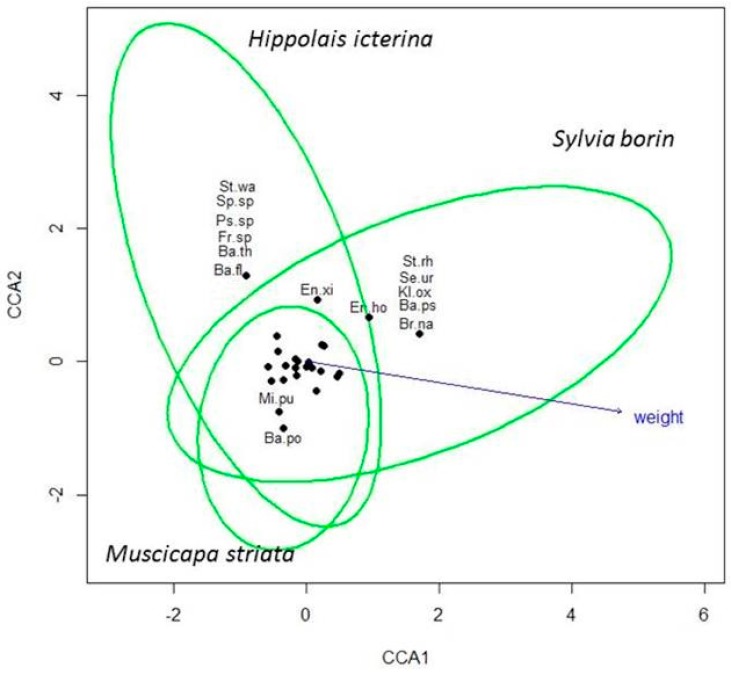
Results of the canonical correspondence analysis (CCA). St.wa = *Staphylococcus warneri*; Sp.sp = *Sphingomonas* sp; Ps.sp = *Pseudomonas* sp.; Fr.sp = *Frondihabitans* sp; Ba.th = *Bacillus thuringiensis*; Ba.fl = *Bacillus flexus*; Ba.ar = *Bacillus megaterium*; En.xi *= Enterobacter xiangfangensis*; En.ho *= Enterobacter hormaechei*; St.rh *= Stenotrophomonas rhizophila*; Se.ur *= Serratia ureilytica*; Kl.ox *= Klebsiella oxytoca*; Ba.ps *= Bacillus pseudomycoides*; Br.na *= Brevundimonas nasdae*; *Mi.pu = Microbacterium pumilum*; Ba.po = *Bacillus pocheonensis.*

The CCA returned a total contingency coefficient of 4.63, of which the species constraint explained a proportion of 0.06 (Figure 3). Therefore, the 0.94 of the total variance was unconstrained to the species. However, the permutation test indicated that only the first constrained axis was significant (*F* = 2.30; *p* = 0.001). The variable that best fit the ordination was bird body mass, which resulted in a high vector dimension (0.99) and correlation coefficient (*r^2^* = 0.45; *p* < 0.001).

As reported in Figure 3, some bacteria seem to colonize a particular host in preference to others. For example, *Staphylococcus warneri*, *Sphingomonas* sp., *Pseudomonas* sp., *Frondihabitans* sp., *Bacillus thuringiensis*, and *Bacillus flexus* were found exclusively on *Hippolais icterina*. Similarly, *Stenotrophomonas rhizophila, Serratia ureilytica, Klebsiella oxytoca, Bacillus pseudomycoides*, and *Brevundimonas nasdae* were isolated only from samples belonging to *Sylvia borin* and *Bacillus pocheonensis* on *Muscicapa striata*.

## 4. Discussion

The results confirmed the possibility of the transfer of bacteria, including pathogens, through bird migration between very distant countries. The two tested sampling protocols showed some differences in bacterial isolation performance, but neither procedure was significantly better than the other—some species were detected by both of them while some only by one. This could be due to different affinities between bacterial species for feathers, body position, sampling procedures, or instrumentation; thus, bacterial detectability is affected by the choice of sampling protocol.

In agreement with previous studies, we found a small number of species with a low biodiversity on sampled surfaces [30]. Feathers can be a natural barrier that prevents skin infections caused by bacteria [31]. Birds have evolved several mechanisms to overcome the disadvantages caused by bacteria. For example, to prevent bacteria-induced degradation of feathers [16], uropygium secretions, in addition to lubricating feathers, also attack and inhibit bacteria [32]. However, some bacteria show a strict association with specific hosts, while others appear generalists [33]. Migrant species can also have a lower microbial diversity than residents [19]. Accordingly, in the current study, while two bird species, *Sylvia borin* and *Hippolais icterina,* demonstrated specific bacterial associations, the third one, *Muscicapa striata* did not. One important determinant of bacterial association in birds is their molting strategy, which has been suggested as an adaptation to microbial control [20,34]. The birds sampled in our study molted all feathers on their African wintering grounds, affecting the observed bacterial composition. The abundance of the genus *Bacillus* in the analyzed samples suggests that these bacteria are natural inhabitants of plumage [20,35]. *Bacillus cereus* and *Bacillus megaterium*, which have been widely studied by other authors, have the ability to degrade feather keratin [36,37]. In the current study, some species of bacilli were found exclusively on the Icterine warbler. We are aware that this could be an artifact of the small sample size or of the molecular analysis procedure, yet this result may reflect different habits among bird species, since bird habits are known to be an important determinant of the plumage microbial community (e.g., ground vs aerial foragers) [16,20,33]. A large number of the identified species are widely distributed in the natural environment. *Arthrobacter* sp., *Brevibacterium* sp., and *Curtobacterium* sp. are three genera of Gram-positive bacteria that are commonly found in soil [38,39]. *Flavobacterium* is a genus of Gram-negative bacteria, whose members are widely distributed in nature, occurring mostly in soil, sediment, and aquatic ecosystems [40]. The presence of these species on feathers suggests that birds may acquire them directly from the surrounding environment. Some species of the genus *Arthrobacter* have shown the ability to cause urinary tract infections and infective endocarditis in humans and, therefore, are worthy of further investigation [41,42,43,44]. Within the *Curtobacterium* genus, *C. flaccumfaciens* is known to be a colonizer of plants in which it is responsible for illness and damage such as necrotic lesions and wilt. Migratory birds may, therefore, also be vehicles of bacterial infection and promoters of disease in plants [45]. The observed genera *Klebsiella*, *Enterobacter*, and *Serratia,* collectively called coliform bacilli, include Gram-negative opportunistic pathogens, which are responsible for a wide range of infections [30,46]. The genus *Klebsiella* is ubiquitous in nature, commonly found in surface water, sewage, and plants [47], as well as on the mucosal surfaces of mammals (horses, swine). *Klebsiella* species appear to be relatively common on birds and, as opportunistic pathogens, can affect human health by causing several infections that most commonly involve the urinary and respiratory tracts [48,49]. *Enterobacter* is a genus of common bacteria that are found in habitats such as water, sewage, vegetables, and soil [50]. *Enterobacter spp*, as opportunistic pathogens, can cause several human diseases, including respiratory and urinary tracts infections, meningitis, and bacteremia [51]. *Serratia* is a genus isolated in the natural environment from water, soil, and plants [52]. *Serratia* species are opportunistic pathogens that cause pneumonia and bladder or kidney infections in humans and some zootechnical mammals [52]. Our results showed the presence of *Klebsiella* and *Serratia* on the garden warbler only, while the *Enterobacterium* species were found on both garden and Icterine warblers. Another fact to be emphasized is the finding of the genus *Pantoea* a group of Gram-negative bacteria in all three bird species. The genus is frequently isolated from plant surfaces, seeds, soil, and water [53] and includes many species infective for humans. As reported by Delétoile et al. [54], *P. agglomerans*, which is widely distributed in nature occupying, as preferential habitats, water, soil, and plants, is an opportunistic pathogen that causes numerous cases of infections to soft tissues and bones [55,56,57,58]. Marais et al. [59] also reported on a rare case of cervical spondylodiscitis caused by *P. agglomerans*. Lastly, the discovery of *P. ananti* in the studied samples is of serious concern, in terms of the potential danger to human health (for example, the bacteremic infection case, reported by De Baere et al. [60]) and because it is an emerging pathogen of a large number of agricultural species of commercial importance such as onion, rice, and sudangrass [61,62,63].

The aim of this study was to perform a qualitative analysis of microbial communities living on the chest feathers of migratory birds. The tested protocols showed different affinities for different bacterial species, that is, some bacterial taxa were isolated in all samples taken with RODAC sampling procedure and some were isolated with the Swab sampling procedure. It is, therefore, advisable to use both methods or, more generally speaking, to always use at least two different sampling protocols.

The molecular approach allows easier identification of potentially pathogenic bacteria carried by migratory birds than the microbiological one. The results underline the possible transmission of pathogens during migration, although direct transmission is a rare event, with only a single case found in the review of Tsiodras et al. [64]. We would like to point out that the actual identification of risks may be carried out in the light of the “Epidemiologic Triad”. In reality, factors leading to the outbreak of a disease could be multiple, and a single exposure does not necessarily cause illness. The risk assessment for the manipulation and ringing of migratory birds takes into account three principles: the microbial loads of the bird, the tendency of the operator to contract, and the virulence of the pathogen. Based on our results, the risk of contagious diseases can be considered minimal and potential. Nevertheless, there are certainly numerous considerations which should be made. First, the potential risk for humans and the possibility of the spread of diseases could be accentuated since these birds, during their migration, remain in areas where people live. Second, operators of ringing stations carry out activities for long periods of time and are often in close contact with bird plumage.

It is, therefore, necessary to apply prophylaxis protocols to protect the operators. It would be advisable that those who handle the birds in the ringing station use separate bags to contain the birds, without mixing clean and used ones. Used bags should be washed as often as is compatible with the logistics of the ringing station. It might also be useful to adopt some personal protective equipment such as gowns and goggles. Gloves and masks are not recommended, because they would compromise the manipulation of ringed birds and the proper assignment of fat and muscle scores. Because of the highlighted risks associated with bird migrations, the existing migratory bird ringing programs represent an important opportunity to monitor those aspects and precociously detect the entry of potentially harmful microorganisms. It is, therefore, desirable that all the ringing stations of migratory birds begin routine collections and analysis of microbiological data.

## Figures and Tables

**Table 1:** Birds species, identification codes, and bacterial sampling protocols adopted. In the sampling protocol column, Ssp = Swab sampling procedure and Rsp = RODAC sampling procedure.

	Species	Identification Code	Sampling Protocol	Number of Total Colonies	Number of Isolates
1	*Muscicapa striata*	Ms.1	Ssp	30	9
2	*Muscicapa striata*	Ms.2	Ssp	8	3
3	*Muscicapa striata*	Ms.3	Ssp	28	8
4	*Muscicapa striata*	Ms.4	Ssp	12	8
5	*Muscicapa striata*	Ms.5	Ssp	26	7
6	*Muscicapa striata*	Ms.6	Ssp	9	1
7	*Muscicapa striata*	Ms.7	Ssp	24	6
8	*Muscicapa striata*	Ms.8	Ssp	34	7
9	*Muscicapa striata*	Ms.9	Ssp	7	2
10	*Muscicapa striata*	Ms.10	Ssp	14	4
11	*Muscicapa striata*	Ms.11	Ssp	26	7
12	*Muscicapa striata*	Ms.12	Ssp	21	5
13	*Muscicapa striata*	Ms.13	Ssp	19	7
14	*Muscicapa striata*	Ms.14	Ssp	14	6
15	*Muscicapa striata*	Ms.15	Ssp	16	2
16	*Muscicapa striata*	Ms.16	Rsp	11	3
17	*Muscicapa striata*	Ms.17	Rsp	19	5
18	*Muscicapa striata*	Ms.18	Rsp	24	10
19	*Muscicapa striata*	Ms.19	Rsp	8	3
20	*Muscicapa striata*	Ms.20	Rsp	12	4
21	*Hippolais icterina*	Hi.1	Ssp	29	8
22	*Hippolais icterina*	Hi.2	Ssp	26	7
23	*Hippolais icterina*	Hi.3	Ssp	21	7
24	*Hippolais icterina*	Hi.4	Ssp	31	7
25	*Hippolais icterina*	Hi.5	Ssp	19	4
26	*Hippolais icterina*	Hi.6	Ssp	15	7
27	*Hippolais icterina*	Hi.7	Ssp	8	2
28	*Hippolais icterina*	Hi.8	Ssp	18	3
29	*Hippolais icterina*	Hi.9	Ssp	21	5
30	*Hippolais icterina*	Hi.10	Rsp	18	5
31	*Hippolais icterina*	Hi.11	Rsp	14	5
32	*Hippolais icterina*	Hi.12	Rsp	10	2
33	*Hippolais icterina*	Hi.13	Ssp	19	4
34	*Hippolais icterina*	Hi.14	Rsp	11	6
35	*Hippolais icterina*	Hi.15	Rsp	27	6
36	*Hippolais icterina*	Hi.16	Rsp	18	4
37	*Hippolais icterina*	Hi.17	Ssp	14	5
38	*Hippolais icterina*	Hi.18	Ssp	31	7
39	*Hippolais icterina*	Hi.19	Ssp	24	7
40	*Hippolais icterina*	Hi.20	Rsp	9	3
41	*Sylvia borin*	Sb.1	Ssp	11	3
42	*Sylvia borin*	Sb.2	Ssp	32	8
43	*Sylvia borin*	Sb.3	Ssp	9	4
44	*Sylvia borin*	Sb.4	Ssp	8	1
45	*Sylvia borin*	Sb.5	Ssp	15	4
46	*Sylvia borin*	Sb.6	Rsp	18	6
47	*Sylvia borin*	Sb.7	Rsp	23	7
48	*Sylvia borin*	Sb.8	Rsp	12	3
49	*Sylvia borin*	Sb.9	Ssp	19	5
50	*Sylvia borin*	Sb.10	Ssp	32	7
51	*Sylvia borin*	Sb.11	Ssp	14	6
52	*Sylvia borin*	Sb.12	Ssp	10	3
53	*Sylvia borin*	Sb.13	Ssp	23	6
54	*Sylvia borin*	Sb.14	Ssp	18	4
55	*Sylvia borin*	Sb.15	Ssp	16	3
56	*Sylvia borin*	Sb.16	Ssp	11	3
57	*Sylvia borin*	Sb.17	Ssp	33	7
58	*Sylvia borin*	Sb.18	Rsp	17	4
59	*Sylvia borin*	Sb.19	Rsp	31	9

**Table 2:** Bacterial species isolated from *Muscicapa striata*.

	Ms.1	Ms.2	Ms.3	Ms.4	Ms.5	Ms.6	Ms.7	Ms.8	Ms.9	Ms.10	Ms.11	Ms.12	Ms.13	Ms.14	Ms.15	Ms.16	Ms.17	Ms.18	Ms.19	Ms.20
***Arthrobacter* sp.**	+	−	−	+	−	−	+	+	+	−	−	−	−	−	−	−	+	+	−	−
***Bacillus cereus***	−	−	−	−	−	−	−	+	−	−	+	−	−	+	−	−	−	−	−	−
***Bacillus endophyticus***	−	−	−	−	−	−	−	−	−	−	+	−	+	−	−	−	−	−	−	−
***Bacillus flexus***	−	−	−	−	−	−	−	−	−	−	−	−	−	−	−	−	−	−	−	−
***Bacillus megaterium***	−	−	−	−	−	−	−	−	−	+	−	−	−	−	−	−	−	−	−	+
***Bacillus pocheonensis***	+	−	−	−	−	−	−	−	−	+	−	−	−	−	−	−	−	−	−	−
***Bacillus pseudomycoides***	−	−	−	−	−	−	−	−	−	−	−	−	−	−	−	−	−	−	−	−
***Bacillus simplex***	−	−	+	−	+	−	+	−	−	+	−	+	−	−	−	−	−	−	−	−
***Bacillus* sp.**	+	+	+	+	+	+	+	−	−	−	+	+	−	+	−	+	+	+	−	+
***Bacillus thuringiensis***	−	−	−	−	−	−	−	−	−	−	−	−	−	−	−	−	−	−	−	−
***Brevibacterium frigoritolerans***	−	−	−	+	−	−	+	−	−	−	+	−	−	−	−	+	−	−	−	−
***Brevibacterium* sp.**	−	−	−	−	−	−	−	−	−	−	−	−	−	−	−	−	−	−	−	−
***Brevundimonas nasdae***	−	−	−	−	−	−	−	−	−	−	−	−	−	−	−	−	−	−	−	−
***Curtobacterium flaccumfaciens***	+	−	+	+	−	−	−	+	+	+	+	+	−	−	−	−	−	+	−	−
***Curtobacterium herbarum***	+	−	+	+	−	−	−	−	−	−	−	−	−	−	−	−	−	−	−	−
***Curtobacterium* sp.**	−	−	+	+	+	−	+	+	−	−	−	−	−	+	+	−	+	+	+	−
***Enterobacter hormaechei***	−	−	−	−	−	−	−	−	−	−	−	−	−	−	−	−	−	−	−	−
***Enterobacter ludwigii***	+	−	−	−	−	−	−	+	−	−	−	−	−	−	−	−	−	+	−	−
***Enterobacter* sp.**	+	−	−	+	−	−	+	−	−	−	+	+	+	+	−	+	+	−	−	−
***Enterobacter xiangfangensis***	−	−	−	−	−	−	−	−	−	−	−	−	−	−	−	−	−	−	−	−
***Flavobacterium* sp.**	−	−	−	+	−	−	−	−	−	−	−	−	−	−	−	−	−	+	−	−
***Frondihabitans* sp.**	−	−	−	−	−	−	−	−	−	−	−	−	−	−	−	−	−	−	−	−
***Janthinobacterium* sp.**	−	−	−	−	−	−	−	−	−	−	−	−	−	−	−	−	−	−	−	−
***Klebsiella michiganensis***	−	−	+	−	−	−	−	+	−	−	−	−	+	−	−	−	−	+	+	−
***Klebsiella oxytoca***	−	−	−	−	−	−	−	−	−	−	−	−	−	−	−	−	−	−	−	−
***Klebsiella* sp.**	−	−	+	−	+	−	−	−	−	−	−	−	+	−	−	−	−	−	−	−
***Massilia* sp.**	−	−	−	−	+	−	−	−	−	−	−	−	+	−	−	−	−	+	−	−
***Microbacterium pumilum***	−	+	−	−	+	−	−	+	−	−	−	−	−	−	−	−	−	+	+	−
***Pantoea agglomerans***	−	+	−	−	−	−	−	−	−	−	−	−	+	−	−	−	+	+	−	+
***Pantoea ananatis***	−	−	−	−	−	−	−	−	−	−	−	−	−	−	−	−	−	−	−	−
***Pantoea* sp.**	+	−	+	−	−	−	+	−	−	−	+	+	+	+	−	−	−	−	−	−
***Pseudomonas* sp.**	−	−	−	−	−	−	−	−	−	−	−	−	−	−	−	−	−	−	−	−
***Serratia ureilytica***	−	−	−	−	−	−	−	−	−	−	−	−	−	−	−	−	−	−	−	−
***Sphingomonas* sp.**	−	−	−	−	−	−	−	−	−	−	−	−	−	−	−	−	−	−	−	−
***Staphylococcus pasteuri***	+	−	−	−	+	−	−	−	−	−	−	−	−	+	+	−	−	−	−	+
***Staphylococcus warneri***	−	−	−	−	−	−	−	−	−	−	−	−	−	−	−	−	−	−	−	−
***Stenotrophomonas rhizophila***	−	−	−	−	−	−	−	−	−	−	−	−	−	−	−	−	−	−	−	−

**Table 3:** Bacterial species isolated from *Hippolais icterina.*

	Hi.1	Hi.2	Hi.3	Hi.4	Hi.5	Hi.6	Hi.7	Hi.8	Hi.9	Hi.10	Hi.11	Hi.12	Hi.13	Hi.14	Hi.15	Hi.16	Hi.17	Hi.18	Hi.19	Hi.20
***Arthrobacter* sp.**	+	+	+	−	−	−	−	−	+	−	−	−	−	−	−	−	−	+	−	−
***Bacillus cereus***	−	−	−	−	+	−	−	−	−	+	−	−	−	+	−	−	+	−	−	−
***Bacillus endophyticus***	+	−	+	−	−	+	−	−	−	+	−	−	−	+	−	−	−	+	−	−
***Bacillus flexus***	−	−	−	+	−	−	−	−	+	−	−	−	−	−	−	−	−	−	−	−
***Bacillus megaterium***	−	−	−	−	−	−	−	−	−	−	−	−	−	−	−	+	−	−	−	−
***Bacillus pocheonensis***	−	−	−	−	−	−	−	−	−	−	−	−	−	−	−	−	−	−	−	−
***Bacillus pseudomycoides***	−	−	−	−	−	−	−	−	−	−	−	−	−	−	−	−	−	−	−	−
***Bacillus simplex***	−	+	−	−	−	−	−	−	−	−	+	−	−	−	+	−	−	−	−	−
***Bacillus* sp.**	+	+	+	+	+	+	+	+	+	−	+	+	+	+	−	−	+	−	+	−
***Bacillus thuringiensis***	+	−	−	−	−	−	−	−	−	−	−	+	−	−	−	−	−	−	−	−
***Brevibacterium frigoritolerans***	−	−	+	−	−	+	−	−	−	−	+	−	+	−	−	−	−	−	−	−
***Brevibacterium* sp.**	−	−	−	−	−	−	−	−	−	−	−	−	−	−	−	−	−	−	−	−
***Brevundimonas nasdae***	−	−	−	−	−	−	−	−	−	−	−	−	−	−	−	−	−	−	−	−
***Curtobacterium flaccumfaciens***	−	−	+	+	+	+	−	−	−	−	−	−	+	−	+	−	−	+	+	−
***Curtobacterium herbarum***	−	−	−	−	−	−	−	−	+	+	+	−	−	−	+	−	+	−	+	−
***Curtobacterium* sp.**	+	−	−	−	−	+	−	+	−	+	−	−	−	−	−	−	−	−	+	−
***Enterobacter hormaechei***	−	−	−	−	−	−	−	−	−	−	−	−	−	−	−	−	−	+	−	−
***Enterobacter ludwigii***	−	−	−	−	−	−	−	−	−	−	−	−	−	+	−	−	−	−	−	−
***Enterobacter* sp.**	−	+	−	−	−	−	−	−	−	−	−	−	−	−	−	−	−	−	−	+
***Enterobacter xiangfangensis***	−	−	−	−	−	−	−	−	−	−	−	−	−	−	−	−	−	+	−	−
***Flavobacterium* sp.**	−	−	−	−	−	−	−	−	−	−	−	−	−	+	−	−	−	−	−	−
***Frondihabitans* sp.**	−	−	−	+	−	−	−	−	−	−	−	−	−	−	−	−	−	−	−	−
***Janthinobacterium* sp.**	−	−	−	−	−	−	−	−	−	−	−	−	−	−	−	−	−	−	−	−
***Klebsiella michiganensis***	−	−	+	+	−	−	−	+	−	−	−	−	−	+	−	−	+	−	−	−
***Klebsiella oxytoca***	−	−	−	−	−	−	−	−	−	−	−	−	−	−	−	−	−	−	−	−
***Klebsiella* sp.**	−	+	−	−	−	−	−	−	−	−	−	−	−	−	−	−	−	+	−	−
***Massilia* sp.**	+	−	−	−	−	−	−	−	−	−	−	−	−	−	+	−	−	−	−	−
***Microbacterium pumilum***	−	−	−	−	−	−	−	−	−	−	+	−	−	−	−	−	−	−	−	−
***Pantoea agglomerans***	+	+	+	+	+	−	−	−	−	+	−	−	−	−	+	+	−	−	−	−
***Pantoea ananatis***	−	−	−	−	−	−	−	−	−	−	−	−	−	−	−	−	−	−	−	−
***Pantoea* sp.**	−	+	−	+	−	−	+	−	−	−	−	−	−	−	+	+	+	−	+	+
***Pseudomonas* sp.**	−	−	−	−	−	+	−	−	−	−	−	−	−	−	−	−	−	−	−	+
***Serratia ureilytica***	−	−	−	−	−	−	−	−	−	−	−	−	−	−	−	−	−	−	−	−
***Sphingomonas* sp.**	−	−	−	−	−	−	−	−	−	−	−	−	−	−	−	−	−	−	+	−
***Staphylococcus pasteuri***	+	−	−	−	−	+	−	−	+	−	−	−	+	−	−	−	−	+	+	−
***Staphylococcus warneri***	−	−	−	−	−	−	−	−	−	−	−	−	−	−	−	+	−	−	−	−
***Stenotrophomonas rhizophila***	−	−	−	−	−	−	−	−	−	−	−	−	−	−	−	−	−	−	−	−

**Table 4:** Bacteria species isolated from *Sylvia borin.*

	Sb.1	Sb.2	Sb.3	Sb.4	Sb.5	Sb.6	Sb.7	Sb.8	Sb.9	Sb.10	Sb.11	Sb.12	Sb.13	Sb.14	Sb.15	Sb.16	Sb.17	Sb.18	Sb.19
***Arthrobacter* sp.**	−	−	−	−	+	+	−	−	−	−	−	+	+	+	+	−	−	−	+
***Bacillus cereus***	−	−	−	−	−	−	−	+	−	−	+	−	−	−	−	−	−	+	−
***Bacillus endophyticus***	−	−	−	−	−	+	−	−	−	−	−	−	−	−	−	−	−	−	−
***Bacillus flexus***	−	−	−	−	−	−	−	−	−	−	−	−	−	−	−	−	−	−	−
***Bacillus megaterium***	−	−	−	−	−	−	−	−	−	−	−	−	−	−	−	−	−	−	−
***Bacillus pocheonensis***	−	−	−	−	−	−	−	−	−	−	−	−	−	−	−	−	−	−	−
***Bacillus pseudomycoides***	−	−	−	−	−	−	−	−	−	+	−	−	−	−	−	−	+	−	−
***Bacillus simplex***	+	−	−	−	−	−	+	−	−	−	−	−	−	−	−	−	−	−	+
***Bacillus* sp.**	+	+	+	−	−	−	+	−	+	−	+	+	−	−	−	+	−	−	+
***Bacillus thuringiensis***	−	−	−	−	−	−	−	−	−	−	−	−	−	−	−	−	−	−	+
***Brevibacterium frigoritolerans***	−	−	−	−	−	−	−	−	−	−	−	−	−	−	−	−	−	−	+
***Brevibacterium* sp.**	−	−	−	−	−	−	−	−	−	−	−	−	−	−	−	−	−	−	−
***Brevundimonas nasdae***	−	−	−	−	−	−	+	−	−	−	−	−	−	−	−	−	−	−	−
***Curtobacterium flaccumfaciens***	−	+	+	+	+	−	−	+	−	−	−	−	−	−	+	−	−	−	+
***Curtobacterium herbarum***	−	−	−	−	+	+	−	−	+	−	+	+	−	−	−	+	+	−	−
***Curtobacterium* sp.**	−	+	+	−	−	−	+	+	−	+	−	−	+	+	−	−	+	−	−
***Enterobacter hormaechei***	−	−	−	−	−	−	−	−	−	+	−	−	−	−	−	−	+	−	−
***Enterobacter ludwigii***	−	−	−	−	−	+	−	−	+	+	−	−	−	−	−	−	+	−	−
***Enterobacter* sp.**	−	+	+	−	−	−	+	−	−	+	−	−	−	−	−	−	−	−	−
***Enterobacter xiangfangensis***	−	−	−	−	−	−	−	−	−	−	−	−	−	+	−	−	−	−	−
***Flavobacterium* sp.**	−	−	−	−	+	−	+	−	−	−	+	−	−	−	−	−	+	−	−
***Frondihabitans* sp.**	−	−	−	−	−	−	−	−	−	−	−	−	−	−	−	−	−	−	−
***Janthinobacterium* sp.**	−	−	−	−	−	−	−	−	−	−	−	−	−	−	−	−	−	+	−
***Klebsiella michiganensis***	−	−	−	−	−	−	+	−	−	−	+	−	+	−	−	+	−	+	−
***Klebsiella oxytoca***	−	−	−	−	−	+	−	−	−	+	−	−	−	−	−	−	−	−	−
***Klebsiella* sp.**	−	+	−	−	−	−	−	−	−	−	+	−	−	−	−	−	−	−	−
***Massilia* sp.**	−	−	−	−	−	−	−	−	−	−	−	−	−	−	+	−	−	−	−
***Microbacterium pumilum***	−	−	−	−	−	−	−	−	−	−	−	−	−	−	−	−	−	−	−
***Pantoea agglomerans***	+	+	−	−	−	+	−	−	+	+	−	−	+	+	−	−	+	+	+
***Pantoea ananatis***	−	−	−	−	−	−	−	−	−	−	−	−	−	−	−	−	−	−	+
***Pantoea* sp.**	−	−	−	−	−	−	−	−	+	−	−	−	+	−	−	−	−	−	−
***Pseudomonas* sp.**	−	−	−	−	−	−	−	−	−	−	−	−	−	−	−	−	−	−	−
***Serratia ureilytica***	−	+	−	−	−	−	−	−	−	−	−	−	−	−	−	−	−	−	−
***Sphingomonas* sp.**	−	−	−	−	−	−	−	−	−	−	−	−	−	−	−	−	−	−	−
***Staphylococcus pasteuri***	−	−	−	−	−	−	−	−	−	−	−	−	+	−	−	−	−	−	+
***Staphylococcus warneri***	−	−	−	−	−	−	−	−	−	−	−	−	−	−	−	−	−	−	−
***Stenotrophomonas rhizophila***	−	+	−	−	−	−	−	−	−	−	−	−	−	−	−	−	−	−	−

**Table 5:** Most abundant genera and species isolated among the bird samples (59).

Genera and Species	Abundance (%)
*Bacillus* sp.	65
*Bacillus cereus*	16
*Bacillus endophyticus*	16
*Bacillus simplex*	18
*Curtobacterium* sp.	38.3
*Curtobacterium flaccumfacies*	41
*Pantoea* sp.	28
*Pantoea agglomerans*	40
*Enterobacter* sp	25
